# Enhanced sensitivity of VEGF detection using catalase-mediated chemiluminescence immunoassay based on CdTe QD/H_2_O_2_ system

**DOI:** 10.1186/s12951-020-00648-9

**Published:** 2020-06-17

**Authors:** Fahimeh Ghavamipour, Hossein Rahmani, Maryam Shanehsaz, Khosro Khajeh, Manouchehr Mirshahi, Reza H. Sajedi

**Affiliations:** 1 Department of Biochemistry, Faculty of Biological Sciences, Tarbiat Modares University, Tehran, 14115-154 Iran; 2 Analytical Chemistry Research Laboratory, Mobin Shimi Azma Company, Tehran, Iran

**Keywords:** Vascular endothelial growth factor (VEGF), Enzyme-linked immunosorbent assays (ELISA), Chemiluminescence, Catalase, Dextran, H_2_O_2_-sensitive quantum dots

## Abstract

**Background:**

Since vascular endothelial growth factor (VEGF) is a significant regulator of cancer angiogenesis, it is essential to develop a technology for its sensitive detection. Herein, we sensitized a chemiluminescence (CL) immunoassay through the combination of H_2_O_2_-sensitive TGA-CdTe quantum dot (QD) as signal transduction, dextran as a cross-linker to prepare enzyme-labeled antigen and the ultrahigh bioactivity of catalase (CAT) as reporter enzyme.

**Results:**

Under the optimized experimental conditions, the chemiluminescence enzyme-linked immunosorbent assay (CL-ELISA) method can detect VEGF in the excellent linear range of 2–35,000 pg mL^−1^, with a detection limit (S/N = 3) of 0.5 pg mL^−1^ which was approximately ten times lower than the commercial colorimetric immunoassay. This proposed method has been successfully applied to the clinical determination of VEGF in the human serum samples, and the results illustrated an excellent correlation with the conventional ELISA method (R^2^ = 0.997). The suitable recovery rate of the method in the serum ranged from 97 to 107%, with a relative standard deviation of 1.2% to 13.4%.

**Conclusions:**

The novel immunoassay proposes a highly sensitive, specific, and stable method for very low levels detection of VEGF that can be used in the primary diagnosis of tumors. With the well-designed sensing platform, this approach has a broad potential to be applied for quantitative analysis of numerous disease-related protein biomarkers for which antibodies are available.
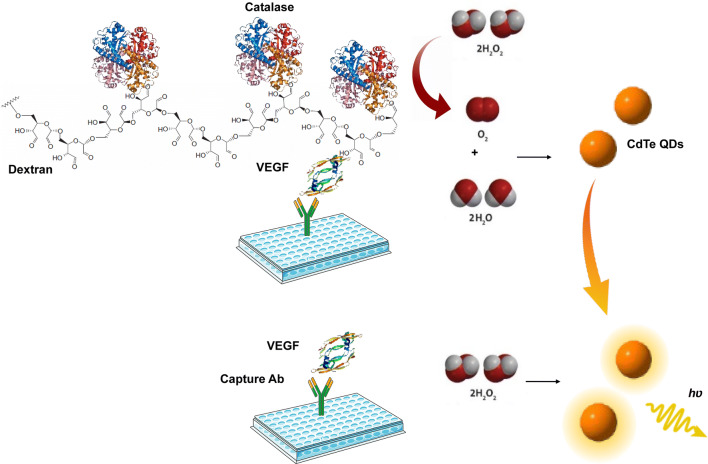

## Background

Accurate detection of tumor markers at low concentration has significant promise for early diagnosis and therapeutic monitoring [1–3]. Vascular endothelial growth factor (VEGF) is a significant regulator of both pathologic and physiologic angiogenesis by activating VEGF-receptor tyrosine kinases in all endothelial cells [4–7]. VEGF is a signaling protein that has been applied as an important serum biomarker for several human diseases, such as cancer [8–11], rheumatoid arthritis [12], psoriasis [13] and proliferating retinopathy [14]. The abnormally rapid growth and division of tumors make it stimulate the overexpression of VEGF due to the supply of more nutrients and oxygen, leading to tumor lymphatic vessels induction and cancer metastasis [15]. It is estimated that up to 60% of human cancer cells overexpress VEGF to create the essential vascular network to support tumor growth and metastasis [16]. A high concentration of VEGF in the tumor tissue and serum of patients who suffer brain tumor was previously reported [17]. Increased VEGF concentration is also highly correlated with tumor progression and survival in patients with malignant melanoma [18]. According to previous researches, the VEGF level in healthy people is usually less than 100 pg mL^−1^ and clearly increased by the progression of the clinical stage (198 pg mL^−1^ in stage I–II; 955 pg mL^−1^ in stage III–IV) in ovarian cancer [19]. Moreover, overexpressed VEGF values were previously reported in patients with brain tumors [20]. Hence, VEGF serum level has important effects as a biomarker on some diseases and subsequent monitoring of treatment.

Avastin, a humanized anti-VEGF antibody, is a potential candidate for the development of anti-angiogenic drugs against VEGF [21, 22] and due to its specificity for VEGF, it is known to be an extremely potent angiogenesis inhibitor [23]. It is a challenging and critical task to develop selective and sensitive detection of VEGF in the patients’ whole blood or serum for early diagnosis of disease and efficient therapeutic monitoring strategies.

VEGF_165_ is the most potent pro-angiogenic isoform and is usually overexpressed in a variety of human tumors [24, 25]. Therefore, the receptor binding domain (RBD) of VEGF_165_ is selected as the target protein in this work. Tumor markers can be detected by different methods such as radioimmunoassay [26], enzyme-linked immunosorbent assays (ELISA) [27], electrophoretic immunoassay [28], and mass spectrometry-based proteomics [29]. ELISA is one of the most common immunoassay methods that is widely used for the detection of different analytes due to its simplicity, rapidity, low cost, robustness, and high-throughput [30]. However, the conventional colorimetric ELISA based on horseradish peroxidase (HRP) displays low sensitivity and fails to meet requirements for higher sensitivity application [31, 32].

Here we improved the detection sensitivity of ELISA by three strategies. At first, catalase (CAT) was used instead of HRP due to its excellent catalytic efficiency. CAT turnover number exceeds any other enzymatic reaction and is estimated to be approximately 4 × 10^7^ [33–36], which is almost 240-fold higher than the one for HRP [37]. The second strategy is to increase the ratio between the enzyme molecule and target analyte at each binding event, which can amplify the detection signal and consequently improves the sensitivity of ELISA. Negatively because of steric reasons in conventional ELISA, a ratio of 1:1 for enzyme and detection antibody is usually used [29, 38]. We use dextran that can address the problem described above as these molecules exhibit three-dimensional and flexible construction with high loading capacity. Meanwhile, to maintain a high CAT activity of CAT–VEGF conjugate to enhance the detection sensitivity, we proposed using the dextran to achieve indirect conjugation of the CAT and VEGF. In addition, due to relatively low color intensity, especially at low analyte concentrations, the signal-generation mechanism based on enzymes that catalyze chromogenic substrates (e.g., tetramethylbenzidine) is not suitable [31, 32]. To enhance the detection sensitivity of conventional ELISA, chromogenic substrates may be replaced by more sensitive signal-generation transducers to convert molecular recognition events into detectable outputs, including chemiluminescent substrates, as the third strategy.

Chemiluminescence enzyme-linked immunosorbent assay (CL-ELISA) is an excellent alternative method for sample screening with the significant merits of high sensitivity, low noise, no interference from background scattering light, broader linearity, reduced assay time, free of radioactive reagents, simple instrument and ease of use. These advantages of CL-ELISA make it a useful detection system. Up to now, several chemiluminescent immunoassay methods have been established in the clinical detection of tumor markers, such as alpha-fetoprotein (AFP), prostate-specific antigen (PSA), carbohydrate antigen 125 (CA125), and neuron-specific enolase (NSH) [39–42].

Quantum dots (QDs) as a family of versatile nanoparticle have shown attractive prospects for the development of novel sensors due to their unique optical properties and effects associated with quantum confinement [43–45]. QDs have been reported as probe for many applications such as pathogenic bacteria [46, 47] and analyte detection [48–51], cancer cell imaging [52], proteomics [53], and others [54, 55]. One considerable property of TGA-CdTe QDs is being sensitive to hydrogen peroxide (H_2_O_2_), leading to the QDs CL. We have recently developed a catalase assay method based on the finding that the CL of the TGA-CdTe/H_2_O_2_ system is reduced owing to the consumption of H_2_O_2_ by the catalytic action of CAT [56]. In the present study, a novel CL based ELISA was developed for the sensitive detection of VEGF, in which H_2_O_2_-sensitive TGA capped CdTe QDs were introduced as a CL signal output, Avastin (anti-VEGF monoclonal antibody) and CAT–VEGF conjugate as the coating antibody and competitive antigen, respectively. The analytical performances of our proposed method, which is assessed based on the validation process using real sample, demonstrated that the CL-ELISA could be applied for selective detection of VEGF molecules in real samples accurately.

## Methods

### Chemicals and materials

Bovine liver catalase (CAT), bovine serum albumin (BSA) and Dextran T500kD were purchased from Sigma Aldrich Chemical Co. (St. Louis, MO, USA). The Ni–NTA agarose was provided by Qiagen (Hilden, Germany). Isopropyl-b-thiogalactopyranoside (IPTG) was purchased from Biobasic Inc. (Canada). The commercial anti-VEGF antibody (Avastin) was purchased from Roche (Switzerland, Basel). H_2_O_2_ (aqueous solution, 30% w/v) and all other chemicals were obtained from Merck (Darmstadt, Germany). Commercial human VEGF ELISA kit was purchased from Abcam (Cambridge, UK). All the other reagents were analytical grade and used directly without further treatments. Ultrapure water was utilized throughout the experiments. Phosphate-buffered saline (PBS, 0.01 M) was prepared by adding 0.2 g KH_2_PO_4_, 2.9 g Na_2_HPO_4_ 12H_2_O, 0.2 g KCl and 8.0 g NaCl into 1000 mL ultrapure water solutions and adjusted to pH 7.0. The washing buffer consisted of 0.05% Tween-20 spiked into phosphate buffer (PBST). The blocking buffer for the residual reactive sites was phosphate buffer containing 0.2% BSA.

### Synthesis of TGA capped CdTe QDs

The TGA-capped CdTe QDs with a certain size, concentration, and maximum emission wavelength (4 nm, 4.0 × 10^−6^ M and 570 nm respectively), as the optimized conditions for the CdTe QDs/H_2_O_2_ CL system, were prepared according to our previous work [56, 57].

### Expression and purification of VEGF

Expression and purification of His-tagged VEGF-RBD [58] (hereinafter would be referred as VEGF) were performed as described previously [59] using pET28a expression vector containing VEGF gene in *E. coli* BL21 cells and Ni–NTA agarose column. Protein expression and purification were evaluated using 12.5% (w/v) sodium dodecyl sulfate polyacrylamide gel electrophoresis (SDS-PAGE) by the method of Laemmli [60] which then stained by Coomassie Brilliant Blue R250. Excess salt in collected fractions was removed by three times dialyzing against PBS containing 10% (v/v) glycerol by gentle stirring for 12 h at 4 °C. Finally, the total VEGF concentration was estimated by the Bradford method, using BSA as the standard [61].

### Preparation of dextran-mediated CAT–VEGF conjugate

The CAT–VEGF conjugate was prepared according to the previous report [57]. In brief, dextran T500kD was activated by 37.5 mg mL^−1^ of periodate in sodium acetate buffer (0.05 M, pH 5.0) at 0 °C for 30 min. Aldehyde production was investigated with 2 mg mL^−1^ of dextran–aldehyde and 2,4-dinitrophenylhydrazine (DNPH, 10%) in 1 M NaOH and formaldehyde was used as a control according to the method of Charbgoo et al. [62, 63]. The CAT–VEGF conjugate was synthesized by suspending CAT, VEGF, and dextran in PBS at a molar ratio of 20: 4: 1. After stirring the mixture in the dark at 10 °C for 72 h, the reactions were stopped by adding 10 μL glycine (2 M). To demonstrate the success of the conjugation reaction, the CAT–VEGF conjugate was characterized by 8% native-PAGE based on the method of Davis [64] which was performed at a constant voltage at 100 V for 120 min at 4 °C and then the gel was stained by Coomassie Brilliant Blue R250.

### Gel filtration

The CAT–VEGF conjugate was separated using Sephadex G-200 (GE Healthcare, Uppsala) gel filtration column equilibrated with 100 mM PB (pH 7.0) at a flow rate of 0.6 mL min^−1^ under the monitoring of A_280_ via an ultraviolet spectrometer. Aliquots of 300 µL of each fraction were collected, and the CAT activity was examined via CL-based CAT assay using H_2_O_2_-sensitive TGA-CdTe quantum dots assay [56]. The protein components of effective fractions were analyzed by 8% native-PAGE and stained by Coomassie Brilliant Blue.

### Optimization of CL-ELISA

Several physicochemical factors that influenced the chemiluminescent ELISA performance were carefully optimized in this work. In order to evaluate the influence of CAT–VEGF conjugate, direct ELISA was performed as follows: The 96-well plates were first coated with 100 μL of anti-VEGF monoclonal antibody (1 μg mL^−1^) in PBS (pH 7.0) and incubated overnight at 4 °C. After washing thrice with PBST, 300 mL of BSA solution (1.0 mg mL^−1^) was used to block the excess sites of the wells. After 2 h of incubation at 37 °C, the microplate was washed with the same procedure. Subsequently, 100 mL of different dilution of CAT–VEGF conjugate in PBS was added into the wells for 2 h at 37 °C. After washing thrice with PBST and once with PBS, 100 μL of 300 mM H_2_O_2_ in 0.01 M PB (pH 7.0) was injected for 1 min. Finally, 100 μL of TGA-CdTe QDs was injected into the well, and the CL signals of the TGA-CdTe QDs were measured by using Berthold luminometer (Titertek-Berthold, Sirius L, Pforzheim, Germany). The effect of the enzyme reaction time on the substrate was also investigated by the assay procedure described above with constant concentration of CAT–VEGF conjugate in different reaction times of 2–120 s.

### Development of direct competitive CL-ELISA for VEGF

The 96-well microplates were modified with 100 μL of anti-VEGF monoclonal antibody (1 μg mL^−1^) in PBS (pH 7.0) at 4 °C overnight, followed washing three times with PBST then blocked with blocking buffer and washed thrice with PBST after 2 h of incubation at room temperature. Subsequently, 100 µL of VEGF standard was added to a desired final concentration up to 50,000 pg mL^−1^ by diluting a stock solution with PBS (0.02 M, pH 7.0). Following the incubation for 1 h at room temperature and washing procedure, 100 μL of CAT–VEGF conjugate was added and incubated in the dark. After 1 h at room temperature, the unbounded content was discarded, and the microplates were washed thrice with PBST and once with PBS. Finally, 100 μL of 300 mM H_2_O_2_ in 0.01 M PB (pH 7.0) per well was injected to the microplates. After the H_2_O_2_ was incubated for 1 min, 100 μL of TGA-CdTe QDs was injected to each well, and the CL signals from the TGA-CdTe QDs, related to the VEGF concentrations, were measured by using luminometer.

### Validation analysis of VEGF sensor

Human serum was threefold diluted with 0.01 M PBS (pH 7.0). Spiked serum samples were prepared by adding the standard VEGF at concentrations of 6000, 220, and 20 pg mL^−1^. Samples were analyzed following the assay procedure described above. To compare them, conventional HRP-based colorimetric immunoassay was conducted by using an ELISA kit according to the operating instructions. The VEGF concentrations were determined using the relevant calibration curves for the CL-ELISA and conventional ELISA assays. All Analyses are always made in triplicate.

## Results and discussion

### Synthesis, characterization and, optimization of TGA-CdTe-QD

The TGA-CdTe QDs were synthesized and characterized as we previously reported [56] by using UV–visible absorption, fluorescence spectra, CL kinetic curve, DLS and, TEM studies. All required details and explanation are presented in Additional file 1: Figure S1. Based on results, we concluded that our TGA-CdTe QDs exhibit uniform size distribution and excellent optical properties. Similar to the previously reported results, we find that the following experimental conditions give the best CL intensity: 800 mM H_2_O_2_, 200 mM NaOH and, size around 4 nm TGA-CdTe QDs as synthesized with a concentration of 4.0 × 10^−6^ M [56].

### Preparation of CAT–VEGF conjugate

pET28a containing VEGF RBD genes was transformed into *E. coli* BL21 competent cells followed by IPTG induction. Protein was efficiently expressed and then purified by Ni–NTA resin. After dialysis against PBS, the VEGF concentration was determined and subjected to non-reducing SDS-PAGE analysis. A sharp band of the purified expected size (~ 28 kDa) was observed by SDS-PAGE (Additional file 1: Figure S2a).

**Fig. 1:**
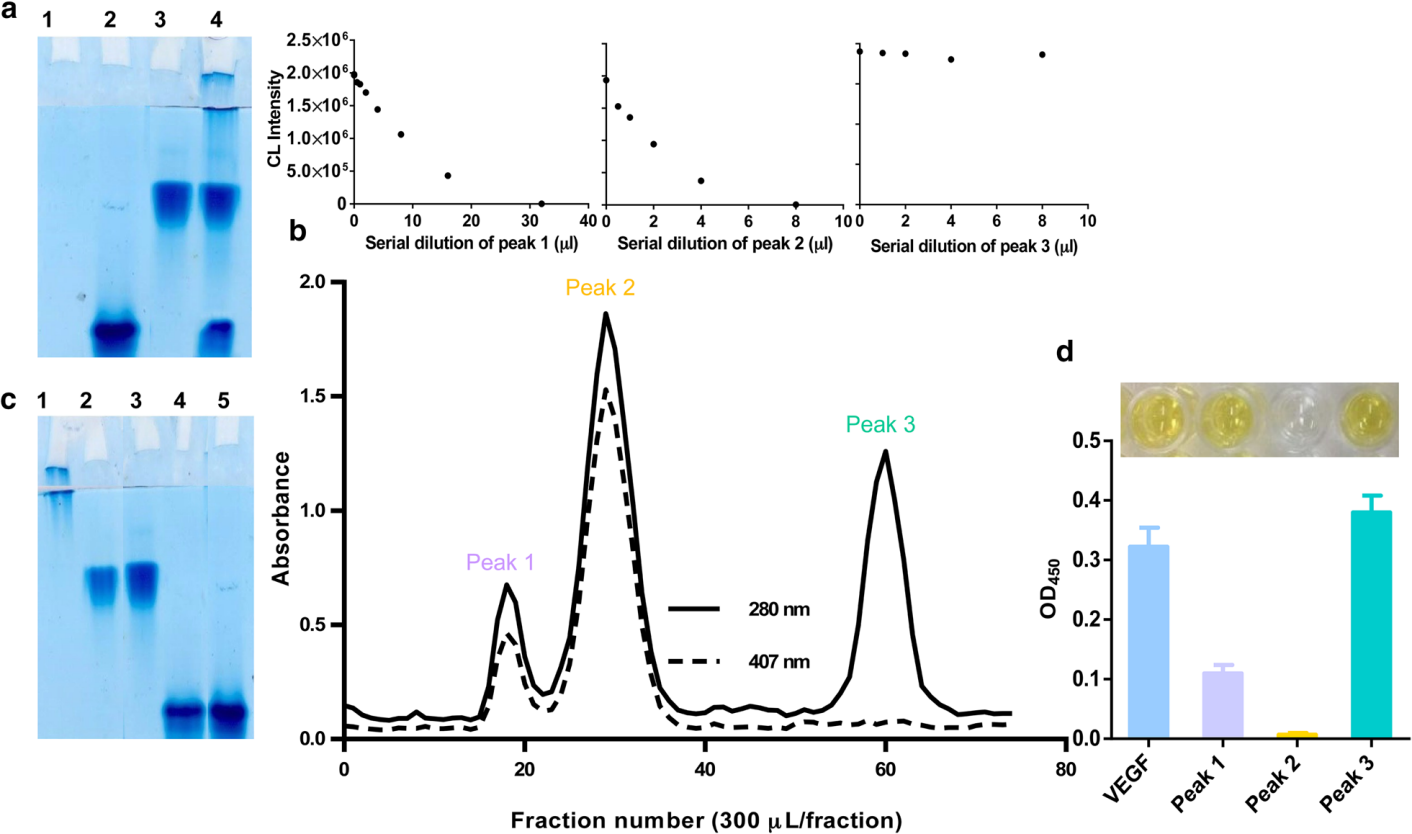
**a** Native-PAGE analysis of dextran-mediated conjugation of CAT with VEGF. Lanes 1, 2, and 3 contain dextran, VEGF and CAT as control, respectively; Lane 4, constitutes the conjugate of CAT–VEGF after incubation time. **b** Gel filtration chromatogram using a Sephadex G-200 column. Elution was performed with PB (pH 7.0) at a flow rate of 0.6 mL min^−1^, and the absorbance of each fraction (300 µL/fraction) was monitored at 280 nm. Inset plots are CAT activity of each peak, which was evaluated via CL-based CAT assay using H_2_O_2_-sensitive TGA-CdTe QDs. **c** Native-PAGE analysis of CAT–VEGF conjugate after purification. Lanes 1 and 3 contain VEGF and CAT as control, respectively; Lanes 2, 4, and 5 correspond to peak 3, 2, and 1, respectively. **d** Colorimetric ELISA results for VEGF detection of each peak (Inset: the corresponding photographs)

Since in competitive ELISA for antigen detection, the antigen in a sample competes with antigen conjugated to a reporter enzyme for limited antibody binding sites [65], here to prepare enzyme-labeled antigen, for developed chemiluminescence competitive ELISA method, a dextran was used as a cross-linker to conjugate the VEGF to CAT. The CAT–VEGF conjugate achieved through the formation of amide bond between the –NH_2_ groups of the proteins and the carboxyl group of dextran. Meanwhile, in competitive adsorption on the activated dextran between CAT and VEGF, the CAT possessed advantages in the competition due to the higher concentration compared to VEGF [with the optimized mole ratio of 20: 4: 1 for CAT, VEGF, and activated dextran, respectively (Additional file 1: Figure S2b)] which increases the sensitivity of the proposed method. To demonstrate the successful preparation of CAT–VEGF conjugate, native-PAGE was carried out. As shown in Fig. 1a, line 4 contains a conjugate in addition to VEGF and CAT.

In order to separate non-conjugated proteins, the CAT–VEGF conjugate was subjected to gel filtration chromatography using a Sephadex G-200 column, and the chromatogram at 280 nm is represented in Fig. 1b. We assumed that the first peak, which definitely has a higher molecular weight, contains CAT–VEGF conjugate, and the second and third peaks contain non-conjugated CAT and VEGF, with a molecular weight of 240 and 28 kDa, respectively. Various experiments were carried out to prove this assumption. The UV–vis spectrum displayed the characteristic absorption peak of the Soret iron (III) heme structure of CAT at 406 nm [66], indicating that the first two peaks contain CAT (Fig. 1b). Meanwhile, CAT activity was examined via CL-based CAT assay using H_2_O_2_-sensitive TGA-CdTe QDs [56]. Among the three peaks, the first two peaks possessing CAT activity were observed on the chromatogram (Fig. 1b). Protein components of each peak were also analyzed by native-PAGE to monitor the formation and separation of the CAT–VEGF conjugate. As shown in Fig. 1c, the first peak only includes the conjugate, the second peak only contains the CAT and the third peak only holds the VEGF. Furthermore, all three peaks were subjected to direct ELISA for VEGF detection. As shown in Fig. 1d, peak 1, and peak 3 contain VEGF. These results demonstrated that CAT and VEGF were covalently attached to the dextran and purified from non-conjugated proteins successfully.

### Optimization of the developed CL assay

**Fig. 2:**
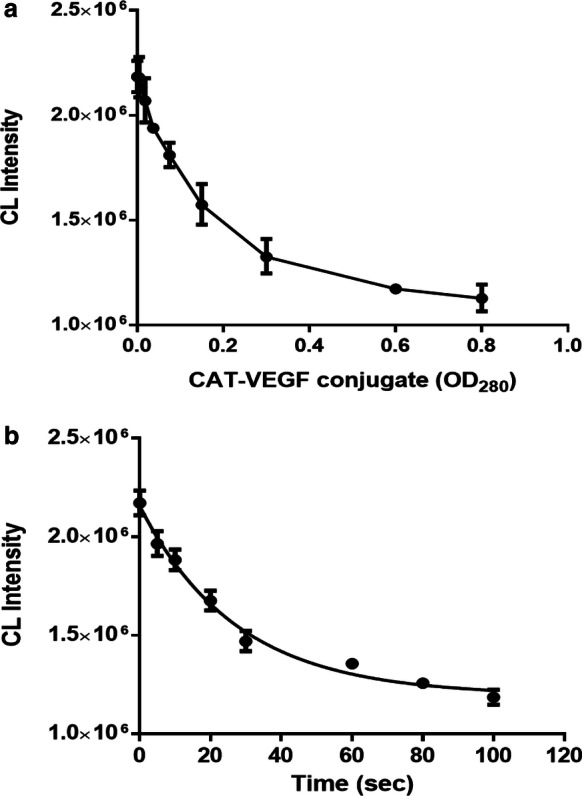
Parameter optimization of the proposed CL-ELISA. **a** CAT–VEGF conjugate concentration. **b** The enzyme reaction time between CAT and H_2_O_2_. The error bars represent the standard deviation of three parallel measurements. Kinetic curves were fitted according to the one-phase decay exponential model of GraphPad Prism version 6 (half-life is 20 s)

Experimental conditions including the concentration of competitive antigen (CAT–VEGF conjugate), H_2_O_2_, and reaction time of CAT, as the most important factors, were investigated to perform developed method under the optimized conditions and improve the immunoassay sensitivity. The concentration of the CAT–VEGF conjugate was optimized to obtain a lower CL response for the positive and higher CL signal for the control sample. Different dilutions of CAT–VEGF conjugate were evaluated by direct ELISA, followed by measuring the corresponding CL intensities of our developed system. The results in Fig. 2a suggests that the CL intensity decreased with increasing the CAT–VEGF conjugate concentration and then reached a minimum value when the OD_280_ was almost 0.3. Therefore, this dilution of CAT–VEGF conjugate was selected as the optimal concentration in subsequent experiments (VEGF and dextran alone, as a control, did not affect CL intensity).

The effect of the enzyme reaction time on the detection sensitivity of the immunoassay experiments obtained by direct ELISA is shown in Fig. 2b. The CL signal decreased as the enzyme reaction time extended, and then reached the plateau when the time prolonged to approximately 60–80 s. Thus, the enzyme reaction time of 60 s was necessary to achieve the highest efficiency for the consumption of H_2_O_2_ by CAT, which is the threefold of half-life (20 s) and the beginning of plateau. Based on our previous reports [56], the alteration in CL of the TGA-CdTe QDs has a good relationship with the concentration of H_2_O_2_ ranging from 0 to 800 mM, demonstrating that the CL of the synthetic TGA-CdTe QDs was extremely sensitive to H_2_O_2_ concentration in solution [56]. To obtain a stable CL signal that is also sensitive enough to H_2_O_2_ reduction, we designated 300 mM H_2_O_2_ as the optimal concentration in the subsequent experiments. Under the optimized concentration of H_2_O_2_, the CL intensity of the QDs would be decreased with the increased CAT concentration due to the decomposition of H_2_O_2_. The previous results indicate that a low amount of CAT was able to cause significant changes in H_2_O_2_ concentration to generate remarkable CL signal fluctuations and subsequently makes the ultrahigh sensitivity of the CL immunoassay.

The following experimental conditions are found to display the best results: a capture antibody concentration of 1.0 μg mL^−1^, H_2_O_2_ concentration of 300 mM, CAT–VEGF conjugate at OD_280_ = 0.3 and enzyme reaction time of 60 s.

### Development of direct competitive CL-ELISA based on H_2_O_2_-induced CL of QDs

**Scheme 1:**
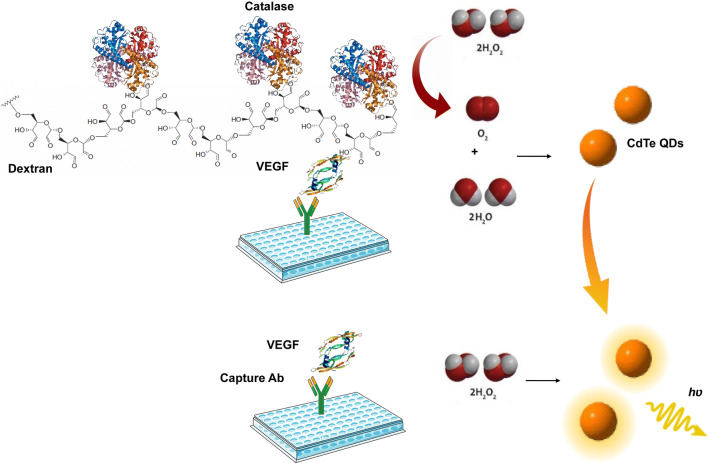
Schematic principle of the CL-ELISA based on H_2_O_2_-sensitive QDs for the detection of VEGF. Anti-VEGF monoclonal antibody was employed as the capture antibodies. Dextran was designed as a bridge to connect the VEGF and CAT, and H_2_O_2_-sensitive TGA-CdTe QDs were used as CL signal output

The schematic illustration of the proposed competitive CL-ELISA is outlined in Scheme 1. The CAT–VEGF conjugate was used as a competitive antigen, and the QD/H_2_O_2_ system was used as the signal transduction of ELISA. The CAT–VEGF conjugate was captured by the anti-VEGF monoclonal antibody pre-coated on the 96-well plate while detecting negative samples. CAT on the conjugate would consume H_2_O_2_. The remaining H_2_O_2_ could trigger QDs, thereby induces the CL signal. Conversely, less amount of CAT–VEGF conjugate was captured when dealing with VEGF-positive samples; hence a low amount of H_2_O_2_ was consumed. More H_2_O_2_ led to a stronger CL of TGA-CdTe QDs, and a higher CL signal was obtained. Therefore, recording the change in CL signals would permit the detection of analytes (Scheme 1).

### VEGF detection using CL-ELISA

**Fig. 3:**
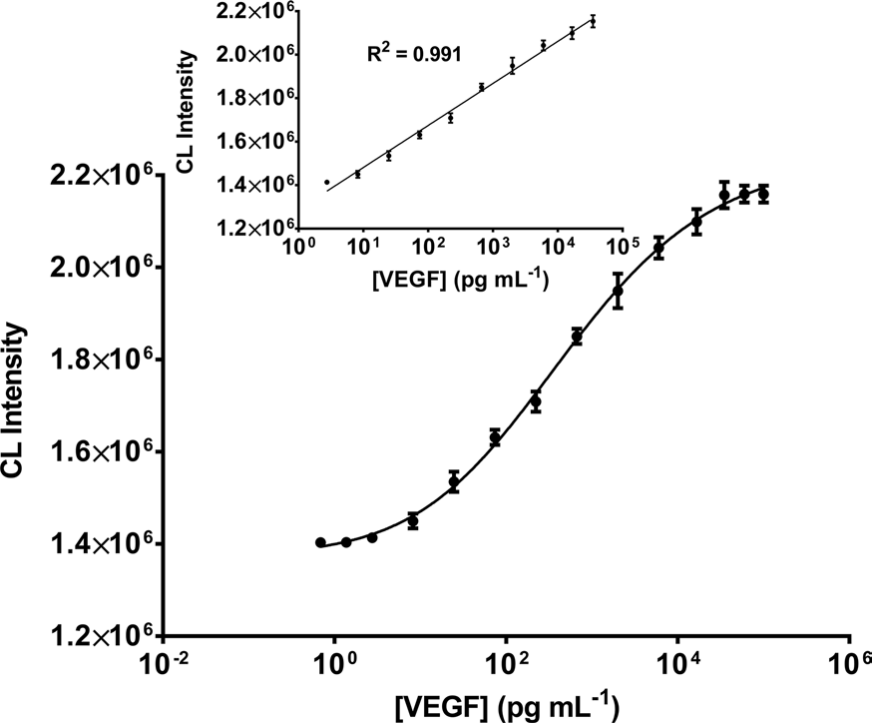
A quantitative immunoassay of VEGF using the developed CL-ELISA. The inset shows a dynamic linear range of VEGF concentrations from 2 to 35,000 pg mL^−1^. The error bars represent the standard deviation of three measurements

Under the optimized conditions, a direct competitive CL-ELISA curve was established. The CL intensity was plotted against various concentrations of the VEGF standard solution (up to 50,000 pg mL^−1^) (Fig. 3). The calibration curve of the developed CL-ELISA exhibited a superior linear range from 2 to 35,000 pg mL^−1^ with a reliable correlation coefficient (R^2^ = 0.991). The regression equation is represented by CL intensity = 193,321 [VEGF] (pg mL^−1^) + 1.868 × 10^6^. In this calibration curve, the limit of detection (LOD) defined as the lowest assayed concentration of analyte, which yields a signal higher than three times the standard deviation of the blank [67] is 0.5 pg mL^−1^. This value is tenfold lower than the conventional HRP-based ELISA (5 pg mL^−1^) and possesses the highest sensitivity and low background signal. Meanwhile, the Kd of 195 pg mL^−1^ for the CAT–VEGF conjugate was calculated from a binding saturation curve (Additional file 1: Figure S3). These results validate the utilization of CL-ELISA for the identification of serum VEGF at low concentrations. The excellently low LOD is attributed to the high efficiency of the CAT, dextran-mediated conjugation, and the use of CL signal of TGA-CdTe QDs.

Furthermore, the performance of the current CAT-mediated CL Immunoassay based on CdTe QD/H_2_O_2_ system was compared with those obtained using other nanomaterial or chemiluminescence-based sensors for VEGF as listed in Table 1. It can be seen that the present sensor exhibited a finer LOD.

**Table 1:** Functional nanomaterials or chemiluminescence -based probes for VEGF detection

Principle	LOD (pg mL^−1^)	Refs.
An electrochemical biosensor based on graphene oxide/ssDNA/PLLA nanoparticles	~ 50	[68]
The AR/H_2_O_2_–Apt–Au NPs/BiOCl nanocomposites probe	~ 25,000	[69]
Magnetic graphene oxide (MGO)-modified Au electrode	~ 31	[70]
Aptamer sandwich based chemiluminescence assay	~ 1000	[71]
Aptamer—gold nanoparticle assembly	~ 45,000	[72]
Gold nanoparticles and immunoreaction using resonance light scattering	~ 60	[73]
Single-step nanoplasmonic aptasensor	~ 25	[74]
CAT-mediated CL immunoassay based on CdTe QD/H_2_O_2_ system	~ 0.5	This study

### Validation studies

**Fig. 4:**
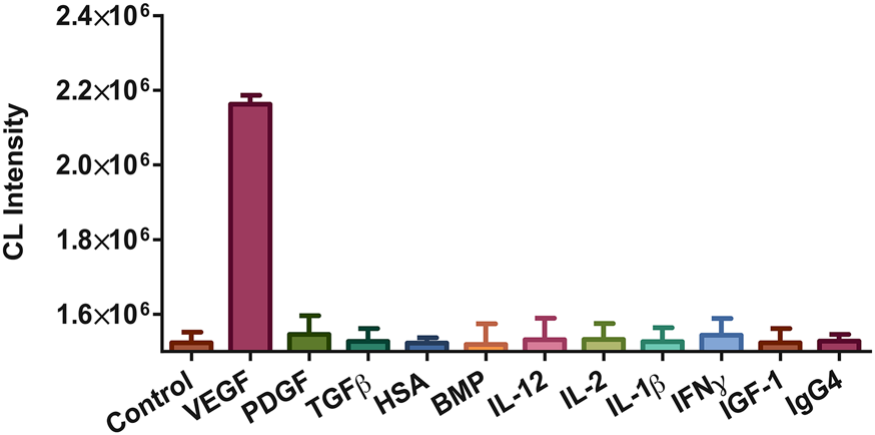
Specificity of the proposed CL-ELISA assay for VEGF detection over other interfering substance. Each independent experiment was repeated three times. VEGF, PDGF, TGFβ, HAS, BMP, IL-12, IL-2, IL-1β, IFNγ, IGF-1, and IgG4 samples were all prepared in a concentration of 50,000 pg mL^−1^. A negative control test was performed by adding PBS

The specificity was determined by evaluating the cross-reaction of the proposed method with 10 common interfering substances in human serum, including platelet-derived growth factor (PDGF), transforming growth factor β (TGFβ), human serum albumin (HSA), bone morphogenetic protein (BMP), interleukin 12 (IL-12), IL-2, IL-1β, Interferon γ (IFNγ), Insulin-like growth factor-1 (IGF-1) and Immunoglobulin G4 (IgG4). In comparison with the negative control, a higher CL was observed when the target VEGF concentration was 50,000 pg mL^−1^ while no significant differences of response signals were observed when other non-specific proteins were detected at the same concentrations (Fig. 4). This finding demonstrates that our strategy has a high selectivity toward the target and those interfering species would not affect the accuracy of results in the clinical diagnosis determination.

To evaluate the accuracy and precision of the proposed detection system, recovery assay was conducted in three replicates of VEGF-spiked serum samples at theoretical concentrations of 6000, 220, and 20 pg mL^−1^ and the increase in the measured concentration of VEGF was determined by comparison to normal serum. Resulting levels in the clinical specimens were then quantified using the calibration curve. The calculated average recovery value was in the range of 76.5–96.7%, with the relative standard derivation of 4.88–16.4%, thereby indicates good accuracy, high reproducibility, and precision of the developed CL-ELISA for quantitative detection of VEGF in actual serum samples. The results also indicated no interference of complex matrices on the developed strategy.

The storage stability of the developed CL immunoassay was studied over a period of one month (data not shown). Prepared devices were kept at 4 °C under dark condition and then were used to measure the current response after 1 month. We see no obvious variations in the detection system. Therefore, the stability was confirmed to be acceptable.

**Fig. 5:**
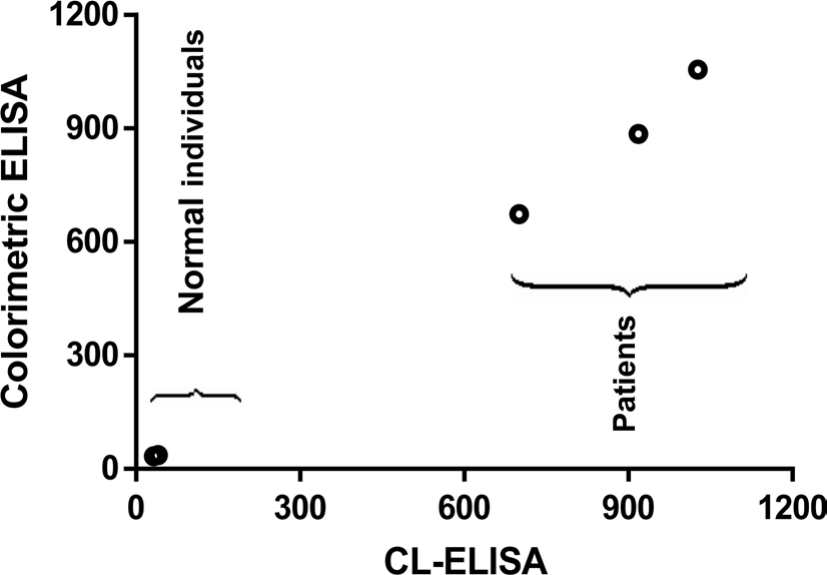
Correlation of the CL-ELISA and conventional colorimetric ELISA results for VEGF detection in 5 serum samples (y = 0.9988x − 6.212, R^2^ = 0.997). Each point represents an average of three replicates

Finally, the reliability of analytical performance and practical value of the developed method was established by comparing the results with those of colorimetric immunoassay kit obtained from blindly detecting five real serum samples. Each sample was analyzed in triplicate, and levels were determined by using the calibration curve. The data obtained from our method were compared with the result by the conventional ELISA method. The concentrations of VEGF in the serum of normal individuals were relatively low compared to the breast, uterus, and brain cancers patients’ serum samples. The relationship between the two methods was assessed by linear regression analysis, and the result was shown in Fig. 5. The two methods exhibited good agreement with a highly significant correlation value (Y = 0.9988x − 6.212, R^2^ = 0.997). The x-axis represented the concentrations of VEGF detected by CL-ELISA, and the y-axis was the concentrations of VEGF detected by colorimetric ELISA. The results indicated high consistency, which reveals that the developed method could be reliable and well suited as a quantitative tool for the analysis of VEGF and even other tumor biomarkers in clinical diagnosis.

## Conclusion

In this study, we demonstrated a novel convenient CL-sensing protocol to detect VEGF by using a competitive immunoreaction strategy. Through the incorporation of three signal amplification factors, including H_2_O_2_-sensitive TGA-CdTe QDs as a CL signal output, dextran-mediated conjugation and ultrahigh catalytic activity of CAT to H_2_O_2_, the proposed immunoassay exhibited high sensitivity for detection of VEGF with an LOD value at 0.5 pg mL^−1^, which was tenfold lower than the conventional ELISA. The analytical performances of our developed method were evaluated in term of accuracy, precision, specificity, and practicability using VEGF-spiked and patient serum samples. The results demonstrated that our proposed CL-ELISA could be applied for the ultrasensitive detection of VEGF in actual serum samples. Furthermore, the results obtained from the proposed method showed a significant correlation with the findings from the confirmatory method. Taking into account of the low detection limit, wide linear range, high reproducibility, excellent specificity, good accuracy, suitable practicability and stability of the immuno sensor, it can be attempted to use for highly sensitive detection of VEGF and numerous analytes, for which antibodies are available, in clinical diagnostics.

## Supplementary information

**Additional file 1. **Figure S1 Characterization of CdTe QDs. (a) UV– visible absorption (―) and fluorescence spectra (---), (b) CL kinetic curves, (c) DLS diagrams and (d) TEM images of CdTe QDs quantum dots with maximum emission peak of 570 nm and average size and concentration of 4 nm and 4.0 × 10^−6^ M, respectively. Figure S2 (a) SDS–PAGE analysis of purified VEGF on a 12.5% gel, stained with Coomassie Brilliant Blue R250. Lane 1: eluted fraction from the Ni-NTA agarose column and lane 2: protein molecular weight marker. (b) Native–PAGE of CAT:VEGF:dextran conjugates formation. The ratios corresponding to each lane are shown at the top of the figure. Figure S3 A dose-response curve of CdTe QD/H_2_O_2_ CL-ELISA against different concentrations of VEGF. Quantitative estimation of Kd value was obtained by non-linear curve fitting (one site binding model) of the data using GraphPad Prism.

## Data Availability

All data generated or analyzed during this study are included in this published article.

## Abbreviations

VEGF: Vascular endothelial growth factor
CL: Chemiluminescence
QD: Quantum dot
CAT: Catalase
ELISA: Enzyme-linked immunosorbent assays
RBD: Receptor binding domain
HRP: Horseradish peroxidase
AFP: Alpha-fetoprotein
PSA: Prostate-specific antigen
CA125: Carbohydrate antigen 125
NSH: Neuron-specific enolase
IPTG: Isopropyl-b-thiogalactopyranoside
PBS: Phosphate-buffered saline
SDS-PAGE: Sodium dodecyl sulfate polyacrylamide gel electrophoresis
BSA: Bovine serum albumin
DNPH: 2,4-Dinitrophenylhydrazine
PDGF: Platelet-derived growth factor
TGFβ: Transforming growth factor β
HAS: Human serum albumin
BMP: Bone morphogenetic protein
IL-12: Interleukin 12
IFNγ: Interferon γ
IGF-1: Insulin-like growth factor-1
IgG4: Immunoglobulin G4

## References

[1] Giljohann DA, Mirkin CA (2009). Drivers of biodiagnostic development. Nature.

[2] Wulfkuhle JD, Liotta LA, Petricoin EF (2003). Early detection: proteomic applications for the early detection of cancer. Nat Rev Cancer.

[3] Kulasingam V, Diamandis EP (2008). Strategies for discovering novel cancer biomarkers through utilization of emerging technologies. Nat Rev Clin Oncol..

[4] Wang HU, Chen Z-F, Anderson DJ (1998). Molecular distinction and angiogenic interaction between embryonic arteries and veins revealed by ephrin-B2 and its receptor Eph-B4. Cell.

[5] Vikkula M, Boon LM, Iii KLC, Calvert JT, Diamonti AJ, Goumnerov B (1996). Vascular dysmorphogenesis caused by an activating mutation in the receptor tyrosine kinase TIE2. Cell.

[6] Ferrara N, Gerber H-P, LeCouter J (2003). The biology of VEGF and its receptors. Nat Med.

[7] Langer I, Vertongen P, Perret J, Fontaine J, Atassi G, Robberecht P (2000). Expression of vascular endothelial growth factor (VEGF) and VEGF receptors in human neuroblastomas. Med Pediatr Oncol.

[8] Carmeliet P, Jain RK (2000). Angiogenesis in cancer and other diseases. Nature.

[9] Plate KH, Breier G, Weich HA, Risau W (1992). Vascular endothelial growth factor is a potential tumour angiogenesis factor in human gliomas in vivo. Nature.

[10] Salven P, Orpana A, Joensuu H (1999). Leukocytes and platelets of patients with cancer contain high levels of vascular endothelial growth factor. Clin Cancer Res.

[11] Salven P, Perhoniemi V, Tykkä H, Mäenpää H, Joensuu H (1999). Serum VEGF levels in women with a benign breast tumor or breast cancer. Breast Cancer Res Treat.

[12] Nakahara H, Song J, Sugimoto M, Hagihara K, Kishimoto T, Yoshizaki K (2003). Anti–interleukin-6 receptor antibody therapy reduces vascular endothelial growth factor production in rheumatoid arthritis. Arthritis Rheum Off J Am Coll Rheumatol..

[13] Detmar M (2004). Evidence for vascular endothelial growth factor (VEGF) as a modifier gene in psoriasis. J Invest Dermatol..

[14] Ray D, Mishra M, Ralph S, Read I, Davies R, Brenchley P (2004). Association of the VEGF gene with proliferative diabetic retinopathy but not proteinuria in diabetes. Diabetes.

[15] Loureiro RMB, D’Amore PA (2005). Transcriptional regulation of vascular endothelial growth factor in cancer. Cytokine Growth Factor Rev.

[16] Ghavamipour F, Shahangian SS, Sajedi RH, Arab SS, Mansouri K, Aghamaali MR (2014). Development of a highly-potent anti-angiogenic VEGF8–109 heterodimer by directed blocking of its VEGFR-2 binding site. FEBS J.

[17] Takano S, Yoshii Y, Kondo S, Suzuki H, Maruno T, Shirai S (1996). Concentration of vascular endothelial growth factor in the serum and tumor tissue of brain tumor patients. Cancer Res.

[18] Ugurel S, Rappl G, Tilgen W, Reinhold U (2001). Increased serum concentration of angiogenic factors in malignant melanoma patients correlates with tumor progression and survival. J Clin Oncol.

[19] Li LI, Wang L, Zhang WEI, Tang B, Zhang J, Song H (2004). Correlation of serum VEGF levels with clinical stage, therapy efficacy, tumor metastasis and patient survival in ovarian cancer. Anticancer Res..

[20] Sciacca FL, Ciusani E, Silvani A, Corsini E, Frigerio S, Pogliani S (2004). Genetic and plasma markers of venous thromboembolism in patients with high grade glioma. Clin Cancer Res.

[21] Wu H-C, Huang C-T, Chang D-K (2008). Anti-angiogenic therapeutic drugs for treatment of human cancer. J Cancer Mol.

[22] Wang Y, Fei D, Vanderlaan M, Song A (2004). Biological activity of bevacizumab, a humanized anti-VEGF antibody in vitro. Angiogenesis.

[23] Hsu M-Y, Yang C-Y, Hsu W-H, Lin K-H, Wang C-Y, Shen Y-C (2014). Monitoring the VEGF level in aqueous humor of patients with ophthalmologically relevant diseases via ultrahigh sensitive paper-based ELISA. Biomaterials.

[24] Nonaka Y, Yoshida W, Abe K, Ferri S, Schulze H, Bachmann TT (2012). Affinity improvement of a VEGF aptamer by in silico maturation for a sensitive VEGF-detection system. Anal Chem.

[25] Niu G, Chen X (2010). Vascular endothelial growth factor as an anti-angiogenic target for cancer therapy. Curr Drug Targets.

[26] Goldsmith SJ (1975). Radioimmunoassay: review of basic principles. Seminars in nuclear medicine.

[27] Voller A, Bartlett A, Bidwell DE (1978). Enzyme immunoassays with special reference to ELISA techniques. J Clin Pathol.

[28] Schmalzing D, Nashabeh W (1997). Capillary electrophoresis based immunoassays: a critical review. Electrophoresis.

[29] Hawkridge AM, Muddiman DC (2009). Mass spectrometry–based biomarker discovery: toward a global proteome index of individuality. Annu Rev Anal Chem..

[30] Asensio L, González I, García T, Martín R (2008). Determination of food authenticity by enzyme-linked immunosorbent assay (ELISA). Food Control.

[31] Jiang W, Beier RC, Luo P, Zhai P, Wu N, Lin G (2016). Analysis of pirlimycin residues in beef muscle, milk, and honey by a biotin–streptavidin-amplified enzyme-linked immunosorbent assay. J Agric Food Chem.

[32] Jeon M, Kim J, Paeng K-J, Park S-W, Paeng IR (2008). Biotin–avidin mediated competitive enzyme-linked immunosorbent assay to detect residues of tetracyclines in milk. Microchem J.

[33] Jamieson D, Chance B, Cadenas E, Boveris A (1986). The relation of free radical production to hyperoxia. Annu Rev Physiol.

[34] George P (1949). The effect of the peroxide concentration and other factors on the decomposition of hydrogen peroxide by catalase. Biochem J..

[35] Gao Z, Xu M, Hou L, Chen G, Tang D (2013). Magnetic bead-based reverse colorimetric immunoassay strategy for sensing biomolecules. Anal Chem.

[36] Nicholls P, Fita I, Loewen PC (2000). Enzymology and structure of catalases. Adv Inorg Chem.

[37] Zhang B, Tang D, Goryacheva IY, Niessner R, Knopp D (2013). Anodic-stripping voltammetric immunoassay for ultrasensitive detection of low-abundance proteins using quantum dot aggregated hollow microspheres. Chem Eur J.

[38] Liu Q, Boyd BJ (2013). Liposomes in biosensors. Analyst..

[39] Zhang X, Guo W, Wang Z, Ke H, Zhao W, Zhang A (2017). A sandwich electrochemiluminescence immunosensor for highly sensitive detection of alpha fetal protein based on MoS2-PEI-Au nanocomposites and Au@ BSA core/shell nanoparticles. Sens Actuators B Chem..

[40] Du Toit SA, Bouwhuis J, Matson M, Musaad S, Davidson JS (2010). Comparison of 2 human chorionic gonadotropin assays as tumor markers assays. Clin Chem.

[41] Ma T, Zhang M, Wan Y, Cui Y, Ma L (2017). Preparation of an acridinium ester-labeled antibody and its application in goldmag nanoparticle-based, ultrasensitive chemiluminescence immunoassay for the detection of human epididymis protein 4. Micromachines..

[42] Liu Z, Zhang L, Yang H, Zhu Y, Jin W, Song Q (2010). Magnetic microbead-based enzyme-linked immunoassay for detection of Schistosoma japonicum antibody in human serum. Anal Biochem.

[43] Du J, Yu C, Pan D, Li J, Chen W, Yan M (2010). Quantum-dot-decorated robust transductable bioluminescent nanocapsules. J Am Chem Soc.

[44] Adegoke O, Forbes PBC (2015). Challenges and advances in quantum dot fluorescent probes to detect reactive oxygen and nitrogen species: a review. Anal Chim Acta.

[45] Akshath US, Shubha LR, Bhatt P, Thakur MS (2014). Quantum dots as optical labels for ultrasensitive detection of polyphenols. Biosens Bioelectron.

[46] Abdelhamid HN, Wu H-F (2018). Selective biosensing of Staphylococcus aureus using chitosan quantum dots. Spectrochim Acta Part A Mol Biomol Spectrosc.

[47] Abdelhamid HN, Wu H-F (2013). Probing the interactions of chitosan capped CdS quantum dots with pathogenic bacteria and their biosensing application. J Mater Chem B..

[48] Xu W, Xiong Y, Lai W, Xu Y, Li C, Xie M (2014). A homogeneous immunosensor for AFB1 detection based on FRET between different-sized quantum dots. Biosens Bioelectron.

[49] Liu F, Zhang Y, Ge S, Lu J, Yu J, Song X (2012). Magnetic graphene nanosheets based electrochemiluminescence immunoassay of cancer biomarker using CdTe quantum dots coated silica nanospheres as labels. Talanta.

[50] Wang X, Sheng P, Zhou L, Tong X, Shi L, Cai Q (2014). Fluorescence immunoassay of octachlorostyrene based on Fo¨ rster resonance energy transfer between CdTe quantum dots and rhodamine B. Biosens Bioelectron.

[51] Abdelhamid HN, Wu H-F (2015). Synthesis and multifunctional applications of quantum nanobeads for label-free and selective metal chemosensing. RSC Adv..

[52] Cui Y, Zhang C, Sun L, Hu Z, Liu X (2015). Direct synthesis of CdS nanodots embedded in bovine serum albumin without external sulfur source for cell imaging. RSC Adv..

[53] Chen Z, Abdelhamid HN, Wu H (2016). Effect of surface capping of quantum dots (CdTe) on proteomics. Rapid Commun Mass Spectrom.

[54] Zhou H, Liu J, Zhang S (2015). Quantum dot-based photoelectric conversion for biosensing applications. TrAC, Trends Anal Chem.

[55] Medintz IL, Stewart MH, Trammell SA, Susumu K, Delehanty JB, Mei BC (2010). Quantum-dot/dopamine bioconjugates function as redox coupled assemblies for in vitro and intracellular pH sensing. Nat Mater.

[56] Ghavamipour F, Sajedi RH, Khajeh K (2018). A chemiluminescence-based catalase assay using H 2 O 2-sensitive CdTe quantum dots. Microchim Acta.

[57] Gharaat M, Sajedi RH, Shanehsaz M, Jalilian N, Mirshahi M, Gholamzad M (2017). A dextran mediated multicolor immunochromatographic rapid test strip for visual and instrumental simultaneous detection of Vibrio cholera O1 (Ogawa) and Clostridium botulinum toxin A. Microchim Acta.

[58] Christinger HW, Muller YA, Berleau LT, Keyt BA, Cunningham BC, Ferrara N (1996). Crystallization of the receptor binding domain of vascular endothelial growth factor. Proteins Struct Funct Bioinforma..

[59] Shahangian SS, Sajedi RH, Hasannia S, Jalili S, Mohammadi M, Taghdir M (2015). A conformation-based phage-display panning to screen neutralizing anti-VEGF VHHs with VEGFR2 mimicry behavior. Int J Biol Macromol.

[60] Laemmli UK (1970). Cleavage of structural proteins during the assembly of the head of bacteriophage T4. Nature.

[61] Bradford MM (1976). A rapid and sensitive method for the quantitation of microgram quantities of protein utilizing the principle of protein-dye binding. Anal Biochem.

[62] Charbgoo F, Mirshahi M, Sarikhani S, Abolhassan MS (2012). Synthesis of a unique high-performance poly-horseradish peroxidase complex to enhance sensitivity of immunodetection systems. Biotechnol Appl Biochem.

[63] Wilson R, Spiller DG, Beckett A, Prior IA, Sée V (2010). Highly stable dextran-coated quantum dots for biomolecular detection and cellular imaging. Chem Mater.

[64] Davis BJ (1964). Disc electrophoresis–II method and application to human serum proteins. Ann N Y Acad Sci.

[65] Yorde DE, Sasse EA, Wang TY, Hussa RO, Garancis JC (1976). Competitive enzyme-liked immunoassay with use of soluble enzyme/antibody immune complexes for labeling. I. Measurement of human choriogonadotropin. Clin Chem..

[66] Periasamy AP, Ho Y-H, Chen S-M (2011). Multiwalled carbon nanotubes dispersed in carminic acid for the development of catalase based biosensor for selective amperometric determination of H2O2 and iodate. Biosens Bioelectron.

[67] De La Rica R, Stevens MM (2012). Plasmonic ELISA for the ultrasensitive detection of disease biomarkers with the naked eye. Nat Nanotechnol.

[68] Pan L-H, Kuo S-H, Lin T-Y, Lin C-W, Fang P-Y, Yang H-W (2017). An electrochemical biosensor to simultaneously detect VEGF and PSA for early prostate cancer diagnosis based on graphene oxide/ssDNA/PLLA nanoparticles. Biosens Bioelectron.

[69] Hsu C-L, Lien C-W, Wang C-W, Harroun SG, Huang C-C, Chang H-T (2016). Immobilization of aptamer-modified gold nanoparticles on BiOCl nanosheets: tunable peroxidase-like activity by protein recognition. Biosens Bioelectron.

[70] Lin C-W, Wei K-C, Liao S, Huang C-Y, Sun C-L, Wu P-J (2015). A reusable magnetic graphene oxide-modified biosensor for vascular endothelial growth factor detection in cancer diagnosis. Biosens Bioelectron.

[71] Shan S, He Z, Mao S, Jie M, Yi L, Lin J-M (2017). Quantitative determination of VEGF165 in cell culture medium by aptamer sandwich based chemiluminescence assay. Talanta.

[72] Shukoor MI, Altman MO, Han D, Bayrac AT, Ocsoy I, Zhu Z (2012). Aptamer-nanoparticle assembly for logic-based detection. ACS Appl Mater Interfaces.

[73] Chen Z, Lei Y, Gao W, Liu J (2013). Detection of vascular endothelial growth factor based on gold nanoparticles and immunoreaction using resonance light scattering. Plasmonics..

[74] Cho H, Yeh E-C, Sinha R, Laurence TA, Bearinger JP, Lee LP (2012). Single-step nanoplasmonic VEGF165 aptasensor for early cancer diagnosis. ACS Nano.

