# Supplementary material for: Enhanced sensitivity of VEGF detection using catalase-mediated chemiluminescence immunoassay based on CdTe QD/H2O2 system
Source: J Nanobiotechnology. 2020 Jun 17;18:93. doi: 10.1186/s12951-020-00648-9 (PMC7302009; doi:10.1186/s12951-020-00648-9)
Supplement: Supplementary file 1 — Additional file 1. Figure S1 Characterization of CdTe QDs. (a) UV– visible absorption (―) and fluorescence spectra (---), (b) CL kinetic curves, (c) DLS diagrams and (d) TEM images of CdTe QDs quantum dots with maximum emission peak of 570 nm and average size and concentration of 4 nm and 4.0 × 10^−6^ M, respectively. Figure S2 (a) SDS–PAGE analysis of purified VEGF on a 12.5% gel, stained with Coomassie Brilliant Blue R250. Lane 1: eluted fraction from the Ni-NTA agarose column and lane 2: protein molecular weight marker. (b) Native–PAGE of CAT:VEGF:dextran conjugates formation. The ratios corresponding to each lane are shown at the top of the figure. Figure S3 A dose-response curve of CdTe QD/H2O2 CL-ELISA against different concentrations of VEGF. Quantitative estimation of Kd value was obtained by non-linear curve fitting (one site binding model) of the data using GraphPad Prism. [file 12951_2020_648_MOESM1_ESM.docx]

**Additional file**

**Enhanced Sensitivity of VEGF Detection Using Catalase-Mediated Chemiluminescence Immunoassay Based on CdTe QD/H_2_O_2_ System**

Fahimeh Ghavamipour^1^, Hossein Rahmani^1^, Maryam Shanehsaz^2^, Khosro Khajeh^1^, Manouchehr Mirshahi^1^ and Reza H.Sajedi^1*^

^1^Department of Biochemistry, Faculty of Biological Sciences, Tarbiat Modares University, Tehran 14115-154, Iran

^2^Analytical Chemistry Research Laboratory, Mobin Shimi Azma Company, Tehran, Iran

^*^Corresponding author: Department of Biochemistry, Faculty of Biological Sciences, Tarbiat Modares University, Tehran 14115-154, Iran, Fax/Tel: +98 21 82884717

E-mail: [sajedi_r@modares.ac.ir](mailto:sajedi_r@modares.ac.ir)

**CdTe QDs characterization**

The UV–visible absorption, fluorescence spectra and dynamic CL intensity–time profiles of CdTe QD in aqueous solution are shown in Fig. S1a-c, respectively. The maximum absorption wavelength of CdTe QD was 540 nm, while the Maximum emission wavelengths was located at 570 nm (excited at 340 nm). The narrow emission spectrum demonstrated the high degree of monodispersity of CdTe QD. Moreover, the dynamic CL intensity–time profiles upon injection of CdTe QDs into the H2O2 solution showed that this CL reaction was very rapid and the CL intensity can reach a maximum after ∼200 ms.

According to the Eq. (1-3), the average size and concentration of CdTe QDs were estimated from the absorption and fluorescence spectrum to be about 4 nm and 4.0 × 10^−6^ M, respectively. The size of the synthesized QD and their morphology was also investigated using DLS and TEM. The TEM image together with the DLS analysis showed monodisperse spherical shaped nanoparticles with size around 4 nm for orange emission (Fig. 1E-G). Characterization of the synthesized QDs confirmed their applicability for designing CL-ELISA system.

D = (9.8127 × 10^-7^) × λ^3^ - (1.7147 × 10^-3^) × λ^2^ + (1.0064) × λ - (194.84) Eq. 1

ε = 10043 (D)^2.12.^ Eq. 2

A=ε.c.l Eq. 3


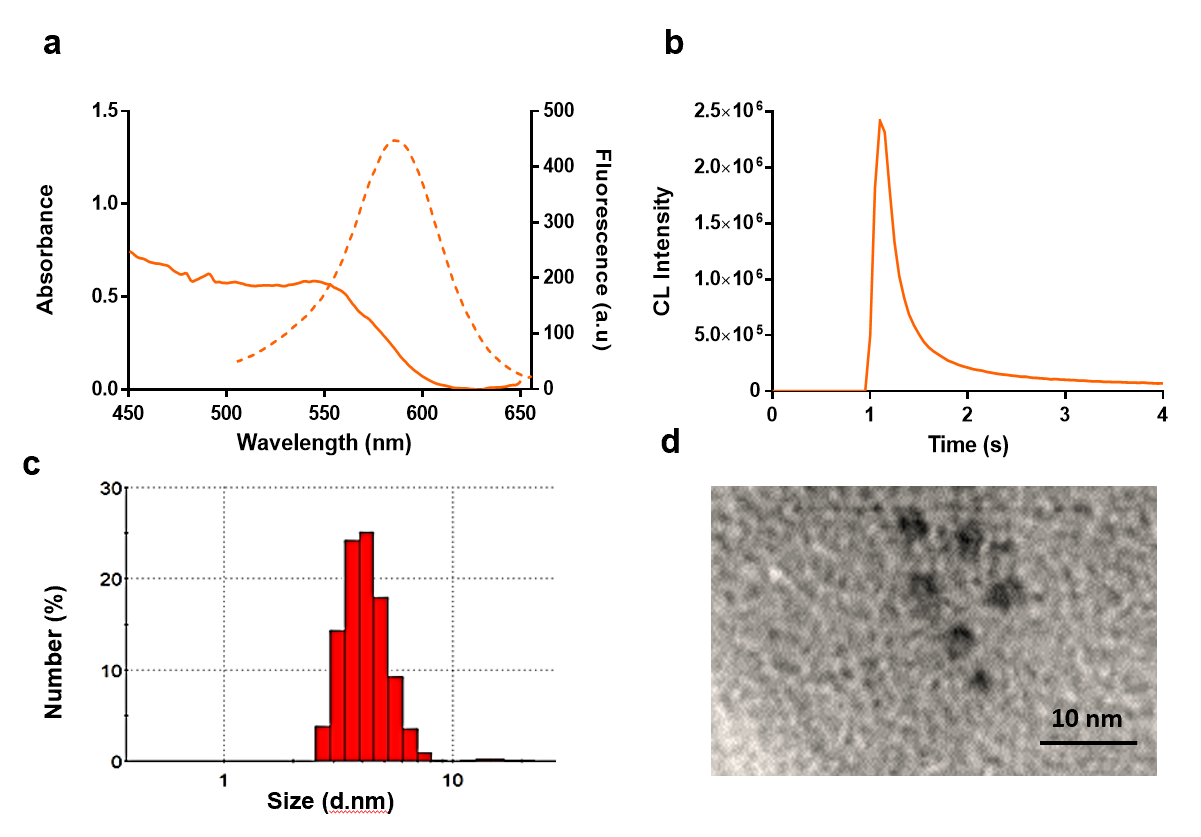


**Figure S1** Characterization of CdTe QDs. (a) UV– visible absorption (―) and fluorescence spectra (---), (b) CL kinetic curves, (c) DLS diagrams and (d) TEM images of CdTe QDs quantum dots with maximum emission peak of 570 nm and average size and concentration of 4 nm and 4.0 × 10^−6^ M, respectively.

**Optimization of the CAT:VEGF:dextran conjugates formation**

After preparation of purified VEGF (Fig.S2a), the oxidized dextran was mixed with different amounts of VEGF and CAT and incubated at 10 °C for 72h in a gentle shaking to obtain the appropriate molar ratios of CAT:VEGF:dextran. Then, the formation of CAT:VEGF:dextran conjugates was checked by using Native–PAGE (Fig. S2b). The results showed that CAT and VEGF was conjugated to the dextran with better efficiency at molar ratios of 80:16:4 or actually the same 20:4:1.


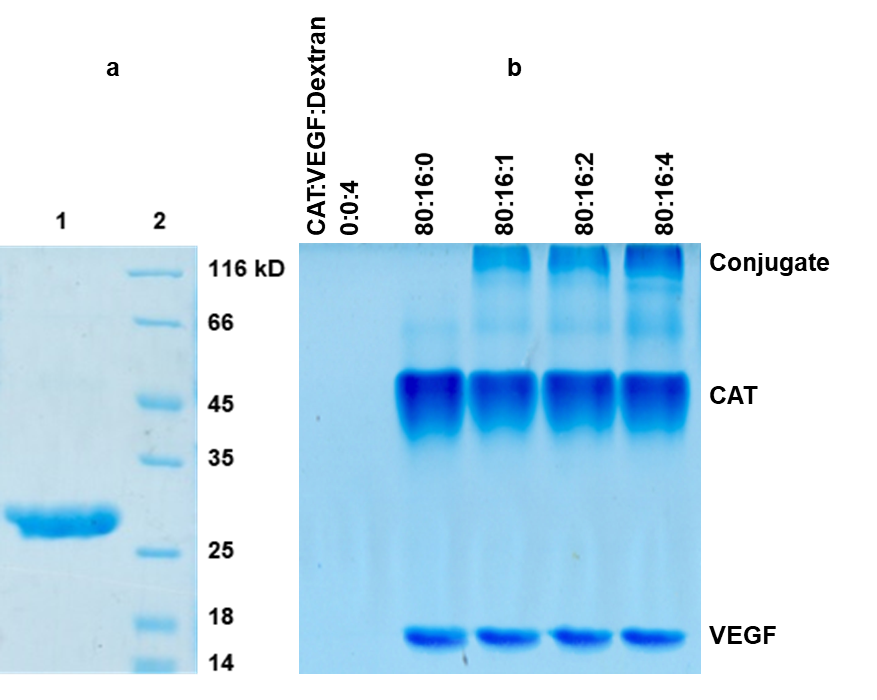


Figure S2 (a) SDS–PAGE analysis of purified VEGF on a 12.5% gel, stained with Coomassie Brilliant Blue R250. Lane 1: eluted fraction from the Ni-NTA agarose column and lane 2: protein molecular weight marker. (b) Native–PAGE of CAT:VEGF:dextran conjugates formation. The ratios corresponding to each lane are shown at the top of the Fig.

**VEGF detection using CL-ELISA**

A binding saturation curve was calculated, and the binding parameters revealed Kd of 195 pg mL^-1^ for the CAT-VEGF conjugate.

Figure S3 A dose-response curve of CdTe QD/H_2_O_2_ CL-ELISA against different concentrations of VEGF. Quantitative estimation of Kd value was obtained by non-linear curve fitting (one site binding model) of the data using GraphPad Prism
